# Supplementary material for: Exposure to hand-held vibrating tools and biomarkers of nerve injury in plasma: a population-based, observational study
Source: BMJ Open. 2023 Jun 30;13(6):e070450. doi: 10.1136/bmjopen-2022-070450 (PMC10314412; doi:10.1136/bmjopen-2022-070450)
Supplement: Author's manuscript [file bmjopen-2022-070450.draft_revisions.pdf]

BMJ Open

BMJ Open is committed to open peer review. As part of this commitment we make the peer review history of every article we publish publicly available.

When an article is published we post the peer reviewers' comments and the authors' responses online. We also post the versions of the paper that were used during peer review. These are the versions that the peer review comments apply to.

The versions of the paper that follow are the versions that were submitted during the peer review process. They are not the versions of record or the final published versions. They should not be cited or distributed as the published version of this manuscript.

BMJ Open is an open access journal and the full, final, typeset and author-corrected version of record of the manuscript is available on our site with no access controls, subscription charges or pay-per-view fees (http://bmjopen.bmj.com).

If you have any questions on BMJ Open's open peer review process please email info.bmjopen@bmj.com

BMJ Open

Exposure to vibrating hand-held tools and biomarkers of nerve injury in plasma - a population-based, observational study

Journal:	*BMJ Open*
Manuscript ID	bmjopen-2022-070450
Article Type:	Original research
Date Submitted by the Author:	24-Nov-2022
Complete List of Authors:	Zimmerman, Malin; Lund University, Department of Translational Medicine; Helsingborg's Hospital, Department of Orthopedics<br>Nilsson, Peter; Lund University, Department of Clinical Sciences Malmö<br>Dahlin, Lars; Lund University, Department of Translational Medicine; Linköping University, Department of Biomedical and Clinical Sciences
Keywords:	Neurophysiology < NEUROLOGY, OCCUPATIONAL & INDUSTRIAL MEDICINE, Neurological injury < NEUROLOGY, Neurological pain < NEUROLOGY

SCHOLARONE™
Manuscripts

I, the Submitting Author has the right to grant and does grant on behalf of all authors of the Work (as defined in the below author licence), an exclusive licence and/or a non-exclusive licence for contributions from authors who are: i) UK Crown employees; ii) where BMJ has agreed a CC-BY licence shall apply, and/or iii) in accordance with the terms applicable for US Federal Government officers or employees acting as part of their official duties; on a worldwide, perpetual, irrevocable, royalty-free basis to BMJ Publishing Group Ltd ("BMJ") its licensees and where the relevant Journal is co-owned by BMJ to the co-owners of the Journal, to publish the Work in this journal and any other BMJ products and to exploit all rights, as set out in our licence.

The Submitting Author accepts and understands that any supply made under these terms is made by BMJ to the Submitting Author unless you are acting as an employee on behalf of your employer or a postgraduate student of an affiliated institution which is paying any applicable article publishing charge ("APC") for Open Access articles. Where the Submitting Author wishes to make the Work available on an Open Access basis (and intends to pay the relevant APC), the terms of reuse of such Open Access shall be governed by a Creative Commons licence – details of these licences and which Creative Commons licence will apply to this Work are set out in our licence referred to above.

Other than as permitted in any relevant BMJ Author's Self Archiving Policies, I confirm this Work has not been accepted for publication elsewhere, is not being considered for publication elsewhere and does not duplicate material already published. I confirm all authors consent to publication of this Work and authorise the granting of this licence.

For peer review only

Exposure to vibrating hand-held tools and biomarkers of nerve injury in plasma - a population-based, observational study

Running head: Vibration exposure and nerve biomarkers

Malin Zimmerman MD, PhD[1,2,3], Peter M. Nilsson, MD, PhD[4], Lars B. Dahlin MD, PhD[1,3,5]*

[1] Department of Translational Medicine – Hand Surgery, Lund University, Malmö, Sweden

[2] Department of Orthopedics, Helsingborg's Hospital, Helsingborg, Sweden

[3] Department of Hand Surgery, Skåne University Hospital, Malmö, Sweden

[4] Department of Clinical Sciences Malmö, Lund University, Malmö, Sweden

[5] Department of Biomedical and Clinical Sciences, Linköping University, Linköping, Sweden

* Corresponding author:

Lars B. Dahlin, Professor, Department of Translational Medicine – Hand Surgery, Lund University, Jan Waldenströms gata 5, 205 02 Malmö, Sweden; Fax +46 40 928855; lars.dahlin@med.lu.se

Word count: 2152

Keyword: Hand-Arm Vibration Syndrome – peripheral nerve - galanin – nerve degeneration –– neuropathy – biomarkers - vibration – HSP27 – caspase 3 – calactin 3 – Nerve Growth Factor

For peer review only - http://bmjopen.bmj.com/site/about/guidelines.xhtml

Abstract

Objectives: To analyze potential biomarkers for vibration-induced nerve damage in a population-based, observational study.

Methods: Neuropathy-relevant plasma biomarkers (VEGF-a, VEGF-d, VEGFR2, galanin, galectin-3 (Gal3), HSP27, ß-NGF, caspase-3, caspase-8, TGF-alpha and TNF) in a sub-cohort of 3,898 individuals (recruited 1991-1996) from Malmö Diet Cancer Study were analyzed during follow up after filling out questionnaires (including question whether work involved work with vibrating hand-held tools; graded as "not at all", "some" or "much").

Results: Among participants, 3361/3898 (86%) reported no work with vibrating hand-held tools, 351/3898 (9%) reported some and 186/3898 (5%) much work. There were more men and smokers in vibration-exposed groups. Galanin levels were higher after much vibration exposure (arbitrary units 5.16±0.71) compared to no without vibration exposure (5.01±0.76; p=0.015) with no other observed differences.

Conclusions: Higher plasma levels of galanin, possibly useful in relation to intensity of vibration, may be found in individuals working with hand-held vibrating tools.

For peer review only - http://bmjopen.bmj.com/site/about/guidelines.xhtml

INTRODUCTION

Hand-arm vibration syndrome (HAVS) is a common condition that may induce injuries to a variety of the tissues in the upper extremity, particularly affecting the peripheral nervous and musculoskeletal systems. Approximately 14% of all employed men and 3% of women in Sweden are exposed to vibrations from hand-held tools for at least 25% of their working time.[1] The amount of induced injury to the individual´s extremity depends on the magnitude, frequency and acceleration of the vibration.[2] Vibration exposure may induce the well-known white finger symptoms, but paresthesia, numbness and impaired sensation in the affected extremity, based on an underlaying nerve injury, are probably more common.[3] To confirm a proper HAVS diagnosis, the treating physician must determine a history of vibration exposure, rule out other explaining conditions, and perform the different clinical assessment methods to detect an impaired function.[4] However, there are no specific simple reported biomarkers that can be measured in the blood to support, for example, signs of a neurological injury. The prevalence of vibration-induced neuropathy in HAVS may extend from 7-79% (average 39%).[3] Such symptoms may be caused by structural changes, e.g., extensive axonal degeneration, as observed in biopsies from the posterior interosseous nerve just proximal to the wrist in men exposed to vibrations.[5] Even data from skin biopsies of fingers in vibration-exposed workers indicate that there is a reduction in content of calcitonin gene-related peptide (CGRP) as well as intraepidermal nerve fiber density observed as staining for PGP-9.5 in the biopsies.[6] The structural changes in the peripheral nerve may also induce an increased susceptibility for peripheral nerve compression syndromes, like carpal tunnel syndrome, CTS,[7] in accordance with the relation between diabetic neuropathy and CTS.[8, 9] The aim of the present observational study was to examine several nerve-related biomarkers, as a sign of neuropathy that can be measured in plasma in a cohort of subjects that were exposed to vibrating hand-held tools.

For peer review only - http://bmjopen.bmj.com/site/about/guidelines.xhtml

METHODS

Study population

The cohort Malmö Diet Cancer Study (MDCS)[10] consists of 30,446 individuals from the southern part of Sweden. The individuals, living in the city of Malmö and born 1926-1945, were invited to participate in the study during 1991 to 1996. The individuals underwent a health examination, assessment of laboratory tests, and filled in a large number of questionnaires regarding cardiovascular risk factors as previously described in detail [10]. The rate of attendance was approximately 41%, of which 60% of the individuals were women. The characteristics of the cohort, and the inclusion criteria for MDCS have previously been described.[10-14] From the large cohort of 28,449 individuals (11,246 men and 17,203 women) that underwent adequate baseline examination, 6,103 individuals were invited to the specific cardiovascular sub-cohort of the MDCS, where 5,540 subjects accepted inclusion for collection of fasting blood samples. The details of the laboratory analysis were recently presented.[15, 16] In short, blood samples were analyzed using Olink Proseek Multiplex proximity extension assay, which uses two antibodies that bind to the target protein.[16] In the final sub-cohort, 4,865 samples were sent for analysis and after internal quality control 4,741 individuals remained for evaluation of biomarker panels. In 843 individuals, data on vibration exposure was missing, resulting in 3,898 included individuals for the present study.

To analyze vibration exposure, the following question was included in participants´ questionnaires: *"Does your work involve working with vibrating hand-held tools?"* Participants graded their exposure to hand-held vibrating tools as "Not at all", "Some" and "Much". During follow-up, the specific laboratory tests as described were performed and the following neuropathy-relevant biomarkers were selected: VEGF-a, VEGF-d, VEGFR2,

For peer review only

galanin, galectin-3 (Gal3), HSP27, ß-NGF, caspase-3, caspase-8, TGF-alpha and TNF. Three of these biomarkers have been used in a recent study related to development of the two nerve compression disorders CTS and ulnar nerve entrapment.[15]

Patient and Public Involvement

There was no patient or public involvement in research in the design, or conduct, or reporting, or dissemination plans of the research, since the research design did not require such involvement.

Statistical analysis

Nominal data are presented as number (%) and were compared using the Chi square test. The biomarkers are presented as mean ± standard deviation (SD) and are expressed as arbitrary units (AU). The Kruskal-Wallis test was used to analyze potential differences between groups with a Bonferroni correction due to the number of biomarkers (eleven biomarkers; i.e., requiring $p<0.004$). In subsequent Mann-Whitney pairwise comparisons, p-values were also adjusted by the Bonferroni correction for multiple testing. Linear regression models were used to study the effect on vibration exposure on levels of galanin (see results section on reason why galanin was selected). Two models were used, one unadjusted (Model 1) and one adjusted for age at baseline, sex, diabetes and smoking. A p-value of <0.05 was considered significant. IBM SPSS Statistics for Mac version 28.0 (SPSS Inc., Chicago, IL) was used for the statistical analysis.

RESULTS

In the total number of individuals, 1,603/3,898 (41%) were men and 2,295/3,898 (59%) were women with a mean age of 57±6 years. At baseline, 3,361/3,898 (86%) reported that they did

For peer review only

not at all work with vibrating hand-held tools, while 351/3,898 (9%) and 186/3,898 (5%) reported that their work involved some or much work with vibrating hand-held tools, respectively. There were more men and more smokers in the groups that were exposed to vibrations. The baseline characteristics of the individuals in this study are presented in Table 1.

Table 1. Biomarkers in plasma in workers reporting no, some or much vibration exposure

	"Does your work involve working with vibrating hand-held tools?"			
	**Not at all (n=3361)**	**Some (n=351)**	**Much (n=186)**	**P-value**
Men n (%)	1215 (36)	236 (67)	152 (82)	**<0.001**
Age (years)	57±6	58±6	57±6	0.23
Smoking n (%)	860 (26)	107 (30)	63 (34)	**0.009**
Diabetes n (%)	661 (20)	74 (21)	32 (17)	0.56
VEGF-a (n=3898)	10.0±0.49	10.0±0.44	10.1±0.45	0.25
VEGF-d (n=3896)	6.74±0.48	6.70±0.48	6.74±0.57	0.27
VEGFR2 (n=3788)	6.86±0.39	6.84±0.39	6.81±0.37	0.27
Galanin (n=3898)	5.01±0.76	5.08±0.77	5.16±0.71	**0.003***
Galectin-3 (Gal3)	5.15±0.42	5.10±.44	5.16±0.37	0.12

For peer review only - http://bmjopen.bmj.com/site/about/guidelines.xhtml

(n=3898)				
HSP27	4.76±0.84	4.79±0.80	4.84±0.78	0.46
(n=3897)				
ß-NGF	1.18±0.39	1.36±0.67	1.12±0.25	0.38
(n=403)				
Caspase-3	10.74±0.95	10.80±0.90	10.79±0.93	0.52
(n=3718)				
Caspase-8	1.58±0.69	1.64±0.72	1.70±0.74	0.10
(n=3865)				
TGF-alpha	1.39±0.34	1.38±0.33	1.36±0.32	0.31
(n=3786)				
TNF	1.33±1.68	1.20±1.95	2.18±3.1	0.79
(n=128)				

Biomarkers presented as mean level of AU: arbitrary units ± standard deviation (SD). Statistical analysis based on Chi-Square (sex, smoking and diabetes) or Kruskal Wallis test; requiring $p<0.004$ (eleven biomarkers). * In subsequent Mann-Whitney pairwise comparisons the statistically significant difference was found between "not at all" and "much" with a p-value of 0.015 adjusted by the Bonferroni correction for multiple tests.

For peer review only

Biomarkers

Observed differences in biomarker plasma levels were small (Table 1). Galanin levels were higher in the group reporting much vibration exposure compared to the group without vibration exposure (Table 1). No other differences among the other analyzed biomarkers were observed. When further analyzing galanin levels, being in the group that reported high vibration exposure predicted higher levels of galanin in the unadjusted model (Table 2). When adjusting for age, sex, smoking and diabetes, no significant predictions could be made in the regression model (Table 2).

For peer review only - http://bmjopen.bmj.com/site/about/guidelines.xhtml

Table 2. Linear regression models of galanin levels in plasma **in workers reporting no, some or much vibration exposure**

	**Model 1**	**Model 2**
No vibration exposure	Reference	Reference
Some vibration exposure	0.06 (-0.02-0.15)	-0.04 (-0.12-0.04)
Much vibration exposure	0.14 (0.03-0.26)	-0.02 (-0.13-0.09)
Age		-0.01 (-0.01- -0.01)
Sex (female reference)		0.41 (0.36-0.46)
Smoking		-0.32 (-0.37- -0.27)
Diabetes		-0.13 (-0.19- -0.08)

Unadjusted B-coefficients with 95% confidence interval. Model 1 unadjusted and model 2 adjusted for age, sex, smoking and diabetes.

DISCUSSION

The present population-based, observational study indicates that individuals with exposure to vibrating hand-held tools in a large cohort of around 30,000 patients, where levels of several biomarkers related to nerves, and possible vibration-induced neuropathy, were available in around 3898 individuals with also information about exposure to vibrating hand-held tools, showed increased levels of plasma galanin. In contrast, other relevant nerve-related biomarkers, including the neuroprotective substance HSP27, which is relevant in diabetic neuropathy[17, 18] and nephropathy,[19] did not show any statistically significant alterations among the vibration-exposed individuals.

Galanin is involved in several biological processes,[16, 20] including nociceptive sensory processing in the spinal cord. Galanin and Neuropeptide Y are consistently upregulated on both gene and protein levels in dorsal root ganglia in rodents with a potential relation to pain after a severe nerve injury;[21-23] a symptom which may also be present among individuals

For peer review only

with HAVS.[24, 25] Experimental studies in rats also indicate that a sciatic nerve compression, which is a less pronounced injury, increases the number of galanin-stained sensory neurons in dorsal root ganglia (DRG) compared to uninjured and contralateral sensory neurons.[26] Furthermore, a complete nerve transection is more efficient than a partial nerve transection or a slight compression injury as an inducer of galanin expression in DRG sensory neurons.[26] Such injuries seem to induce an up-regulation of galanin in mainly small diameter sensory neurons and to a lesser degree in larger neurons.[26] Thus, the extent of induction of galanin expression is related to the severity of nerve injury.[27] Interestingly, the induction of galanin in small diameter neurons in the dorsal root ganglia, which are related to smaller diameter nerve fibers in the sciatic nerve, is notable, since small diameter nerve fibers are reported to be affected by experimental vibration exposure in rat paws.[28] The structural changes seem to occur close to the source of vibration exposure and not more proximally.[28] Whether the present increase in galanin is due to up-regulation of galanin in small or large diameter nerve fibers is not possible to analyze in our cohort. However, our results indicate that a self-reported history of slight or moderate exposure to vibrating hand-held tools is sufficient to provoke a nerve injury that results in increased, and persistent, plasma levels of galanin. On the other hand, it is not surprising that for example beta-NGF did not show any increased plasma levels, since such a substance is only transiently up-regulated after a severe nerve injury (i.e., nerve transection)[29] and vibration-exposure may hence not be reflected by any change in beta-NGF in plasma; which is probably also applicable for some of the other biomarkers. Pro- (e.g., TNF) and anti-inflammatory (TGF-ß) cytokines are expressed after Wallerian degeneration, where the former promotes recruitment of macrophages, and the latter are expressed after macrophage recruitment with a task to attenuate inflammation.[30] The present vibration exposure was not severe enough to induce a nerve injury resulting in higher levels of such cytokines among the participants with much

For peer review only

vibration exposure. Another relevant protein is Galectin-3, which is considered to modulate cell to extracellular matrix interactions, but also as a mediator of signal transduction event on the cell surface as well as a variety of extracellular processes.[31] This multicellular protein was selected to be analyzed in relation to vibration exposure since one suspect it may be increased in view of described structural changes in affected nerves. However, the plasma levels were not different between the participants with and without vibration exposure.

Thus, we did not find any increased plasma levels of the other measured biomarkers. The caspases indicate apoptosis of, for example, Schwann cells that occur in a peripheral nerve after injury in experimental systems.[32] A more severe experimental nerve injury may also induce increased up-regulation of HSP27 locally in the peripheral nerve as well as in sensory neurons.[33] Furthermore, a generalized neuropathy, such as diabetic neuropathy, increases plasma levels of HSP27 with an association between plasma levels and protection of nerve function.[17, 18] However, we did not find any increased plasma levels of these biomarkers in the present cohort. There are also reports that there is a significantly altered expression of insulin growth factor-1 (IGF-1) immunoreactivity in tendons and in nerves after experimental vibration exposure.[34, 35] VEGF and its receptors may also be of relevance since they have been implicated in treatment of nerve regeneration[36] and in diabetic neuropathy.[37] This factor, initially defined as being angiogenetic, is described as relevant in the cross-talk between peripheral nerve components and blood vessels;[38] again being of potential interest in HAVS due to the vascular and neural component in the syndrome. However, even the participants opinion of much vibration exposure was not sufficient to increase the plasma levels. Thus, a number of potentially relevant biomarkers were selected and analyzed, but were not found to be high in the plasma. Recently, low molecular organic biomarkers were analyzed in blood samples in vibration exposed workers using metabolomics, showing

For peer review only

differences in those with vibration-induced white fingers.[39] However, these metabolites were not possible to analyze in the present cohort. The interpretation of the present results is that vibration exposure is not sufficient to constantly increase any up-regulation of a variety of biomarkers, except galanin, in the peripheral nerve that is mirrored in the plasma levels.

Strengths and limitations

The limitations of the study include that we had no detailed data on vibration exposure, such as cumulative exposure (i.e., magnitude, frequency, and acceleration), which would have enabled more detailed analysis. However, the strengths include the well-defined cohort and the large study sample.

CONCLUSIONS

We conclude that higher levels of plasma galanin may be found in individuals reporting work with vibrating hand-held tools. Future research should investigate whether galanin might be useful as a biomarker in HAVS in relation to vibration exposure at various magnitudes, frequencies, and accelerations.

For peer review only

Acknowledgements

The authors are very grateful to all the participants in MDCS whose participation enabled this work. We are also grateful to Anders Dahlin for his help with data extraction and Tina Folker for her administrative assistance.

Author contributorship

A significant contribution was made by all authors stated in this article. MZ , PN and LD designed the study, the first draft was made by MZ and LD and all authors contributed to the interpretation of the data. Finally, all authors reviewed and accepted the final version before publishing.

Competing Interests

Authors declare no conflict of interest.

Funding

This work was supported by grants from Region Skåne [not applicable/NA], Lund University [not applicable/NA], Kockska stiftelsen [not applicable/NA], Stig and Ragna Gorthon Foundation [not applicable/NA], The Swedish Diabetes Foundation [DIA2020-492], the Swedish Research Council [2022-01942], the Regional Agreement on Medical Training and Clinical Research (ALF) between Region Skåne and Lund University [not applicable/NA] and Magnus Bergvall foundation [2020-03612]. The sponsors had no role in the design of the study, collection, analysis, and interpretation of data, writing of the report or submission of the paper.

Data availability statement

For peer review only - http://bmjopen.bmj.com/site/about/guidelines.xhtml

Public access to data is restricted by the Swedish Authorities (Public Access to Information and Secrecy Act; https://government.se/information-material/2009/09/public-access-toinformation-andsecrecy-act/), but data can be made available for researchers after a special review that includes approval of the research project by both the national Swedish Ethical

Ethics

This project has been approved by the Regional Ethical Review Board in Lund, Sweden (51-90, 2009-633, 2011-356, 2014-742, 2015-686, 2015-754, 2016-479) and by the Swedish Ethical Review Authority (2019-01439). All participants gave a signed informed consent to participate in the cohort.

For peer review only

References

1. Swedish Work Environment Authority. The Work Environment Act 2014 [Available from: https://www.av.se/en/work-environment-work-and-inspections/acts-and-regulations-about-work-environment/the-work-environment-act/.
2. Griffin MJ. Measurement, evaluation, and assessment of occupational exposures to hand-transmitted vibration. *Occup Environ Med*. 1997;54(2):73-89.
3. Nilsson T, Wahlstrom J, Burstrom L. Hand-arm vibration and the risk of vascular and neurological diseases-A systematic review and meta-analysis. *PLoS One*. 2017;12(7):e0180795.
4. Shen SC, House RA. Hand-arm vibration syndrome: What family physicians should know. *Can Fam Physician*. 2017;63(3):206-10.
5. Stromberg T, Dahlin LB, Brun A, et al. Structural nerve changes at wrist level in workers exposed to vibration. *Occup Environ Med*. 1997;54(5):307-11.
6. Goldsmith PC, Molina FA, Bunker CB, et al. Cutaneous nerve fibre depletion in vibration white finger. *J R Soc Med*. 1994;87(7):377-81.
7. Dahlin LB, Sanden H, Dahlin E, et al. Low myelinated nerve-fibre density may lead to symptoms associated with nerve entrapment in vibration-induced neuropathy. *J Occup Med Toxicol*. 2014;9(1):7.
8. Thomsen NO, Mojaddidi M, Malik RA, et al. Reduced myelinated nerve fibre and endoneurial capillary densities in the forearm of diabetic and non-diabetic patients with carpal tunnel syndrome. *Acta Neuropathol*. 2009;118(6):785-91.
9. Zimmerman M, Gottsater A, Dahlin LB. Carpal Tunnel Syndrome and Diabetes-A Comprehensive Review. *J Clin Med*. 2022;11(6):1674.
10. Berglund G, Elmstahl S, Janzon L, et al. The Malmo Diet and Cancer Study. Design and feasibility. *J Intern Med*. 1993;233(1):45-51.

For peer review only - http://bmjopen.bmj.com/site/about/guidelines.xhtml

11. Rydberg M, Zimmerman M, Lofgren JP, et al. Metabolic factors and the risk of Dupuytren's disease: data from 30,000 individuals followed for over 20 years. *Sci Rep*. 2021;11(1):14669.

12. Hedblad B, Nilsson P, Janzon L, et al. Relation between insulin resistance and carotid intima-media thickness and stenosis in non-diabetic subjects. Results from a cross-sectional study in Malmo, Sweden. *Diabet Med*. 2000;17(4):299-307.

13. Manjer J, Carlsson S, Elmstahl S, et al. The Malmo Diet and Cancer Study: representativity, cancer incidence and mortality in participants and non-participants. *Eur J Cancer Prev*. 2001;10(6):489-99.

14. Xue L, Borne Y, Mattisson IY, et al. FADD, Caspase-3, and Caspase-8 and Incidence of Coronary Events. *Arterioscler Thromb Vasc Biol*. 2017;37(5):983-9.

15. Bergsten E, Rydberg M, Dahlin LB, et al. Carpal Tunnel Syndrome and Ulnar Nerve Entrapment at the Elbow Are Not Associated With Plasma Levels of Caspase-3, Caspase-8 or HSP27. *Front Neurosci*. 2022;16:809537.

16. Korduner J, Nilsson PM, Melander O, et al. Proteomic and Metabolomic Characterization of Metabolically Healthy Obesity: A Descriptive Study from a Swedish Cohort. *J Obes*. 2021;2021:6616983.

17. Pourhamidi K, Dahlin LB, Boman K, et al. Heat shock protein 27 is associated with better nerve function and fewer signs of neuropathy. *Diabetologia*. 2011;54(12):3143-9.

18. Pourhamidi K, Skarstrand H, Dahlin LB, et al. HSP27 concentrations are lower in patients with type 1 diabetes and correlate with large nerve fiber dysfunction. *Diabetes Care*. 2014;37(3):e49-50.

19. Jakhotia S, Sivaprasad M, Shalini T, et al. Circulating levels of Hsp27 in microvascular complications of diabetes: Prospects as a biomarker of diabetic nephropathy. *J Diabetes Complications*. 2018;32(2):221-5.

For peer review only

20. Ullrich D, Mac Gillavry DW. Mini-review: A possible role for galanin in post-traumatic stress disorder. *Neurosci Lett*. 2021;756:135980.

21. Chalaki M, Cruz LJ, van Neerven SGA, et al. Molecular Changes in the Dorsal Root Ganglion during the Late Phase of Peripheral Nerve Injury-induced Pain in Rodents: A Systematic Review. *Anesthesiology*. 2022;136(2):362-88.

22. Shi TJ, Tandrup T, Bergman E, et al. Effect of peripheral nerve injury on dorsal root ganglion neurons in the C57 BL/6J mouse: marked changes both in cell numbers and neuropeptide expression. *Neuroscience*. 2001;105(1):249-63.

23. Zhang X, Xu ZO, Shi TJ, et al. Regulation of expression of galanin and galanin receptors in dorsal root ganglia and spinal cord after axotomy and inflammation. *Ann N Y Acad Sci*. 1998;863:402-13.

24. Alvarez P, Bogen O, Levine JD. Nociceptor Interleukin 33 Receptor/ST2 Signaling in Vibration-Induced Muscle Pain in the Rat. *J Pain*. 2020;21(3-4):506-12.

25. House R, Wills M, Liss G, et al. Upper extremity disability in workers with hand-arm vibration syndrome. *Occup Med (Lond)*. 2009;59(3):167-73.

26. Dahlin LB, Stenberg L, Kanje M. Galanin expression in sensory neurons after nerve compression or transection. *Neuroreport*. 2003;14(3):359-62.

27. Ma W, Bisby MA. Differential expression of galanin immunoreactivities in the primary sensory neurons following partial and complete sciatic nerve injuries. *Neuroscience*. 1997;79(4):1183-95.

28. Lundborg G, Dahlin LB, Hansson H-A, et al. Vibration exposure and peripheral nerve fiber damage. *J Hand Surg*. 1990;15A:346-51.

29. Shakhbazau A, Martinez JA, Xu QG, et al. Evidence for a systemic regulation of neurotrophin synthesis in response to peripheral nerve injury. *J Neurochem*. 2012;122(3):501-11.

For peer review only

30. Fregnan F, Muratori L, Simoes AR, et al. Role of inflammatory cytokines in peripheral nerve injury. *Neural Regen Res*. 2012;7(29):2259-66.

31. Ochieng J, Furtak V, Lukyanov P. Extracellular functions of galectin-3. *Glycoconj J*. 2002;19(7-9):527-35.

32. Stenberg L, Dahlin LB. Gender differences in nerve regeneration after sciatic nerve injury and repair in healthy and in type 2 diabetic Goto-Kakizaki rats. *BMC Neurosci*. 2014;15:107.

33. Stenberg L, Hazer Rosberg DB, Kohyama S, et al. Injury-Induced HSP27 Expression in Peripheral Nervous Tissue Is Not Associated with Any Alteration in Axonal Outgrowth after Immediate or Delayed Nerve Repair. *Int J Mol Sci*. 2021;22(16).

34. Hansson HA, Dahlin LB, Löwenadler B, et al. Transient increase in insulin-like growth factor 1 immunoreactivity in rat peripheral nerves exposed to vibrations. *Acta Phys Scand*. 1988;132:35-41.

35. Hansson HA, Dahlin LB, Lundborg G, et al. Transiently increased insulin-like growth factor 1 immunoreactivity in tendons after vibration trauma. *Scand J Plast Reconstr Surg Hand Surg*. 1988;22:1-6.

36. Fu C, Hong G, Wang F. Favorable effect of local VEGF gene injection on axonal regeneration in the rat sciatic nerve. *J Huazhong Univ Sci Technolog Med Sci*. 2007;27(2):186-9.

37. Mojaddidi MA, Ahmed MS, Ali R, et al. Molecular and pathological studies in the posterior interosseous nerve of diabetic and non-diabetic patients with carpal tunnel syndrome. *Diabetologia*. 2014;57(8):1711-9.

38. Ruiz de Almodovar C, Lambrechts D, Mazzone M, et al. Role and therapeutic potential of VEGF in the nervous system. *Physiol Rev*. 2009;89(2):607-48.

For peer review only

39. Vihlborg P, Graff P, Hagenbjork A, et al. Serum Metabolites in Hand-Arm Vibration Exposed Workers. *J Occup Environ Med*. 2020;62(7):460-5.

For peer review only

STROBE Statement—checklist of items that should be included in reports of observational studies

	Item No.	Recommendation	Page No.	Relevant text from manuscript
**Title and abstract**	1	(*a*) Indicate the study's design with a commonly used term in the title or the abstract	1	
		(*b*) Provide in the abstract an informative and balanced summary of what was done and what was found	2	
**Introduction**				
Background/rationale	2	Explain the scientific background and rationale for the investigation being reported	3	
Objectives	3	State specific objectives, including any prespecified hypotheses	3	
**Methods**				
Study design	4	Present key elements of study design early in the paper	4	
Setting	5	Describe the setting, locations, and relevant dates, including periods of recruitment, exposure, follow-up, and data collection	4-5	
Participants	6	(*a*) *Cohort study*—Give the eligibility criteria, and the sources and methods of selection of participants. Describe methods of follow-up<br>*Case-control study*—Give the eligibility criteria, and the sources and methods of case ascertainment and control selection. Give the rationale for the choice of cases and controls<br>*Cross-sectional study*—Give the eligibility criteria, and the sources and methods of selection of participants	4-5	
		(*b*) *Cohort study*—For matched studies, give matching criteria and number of exposed and unexposed<br>*Case-control study*—For matched studies, give matching criteria and the number of controls per case		
Variables	7	Clearly define all outcomes, exposures, predictors, potential confounders, and effect modifiers. Give diagnostic criteria, if applicable	4-5	
Data sources/ measurement	8*	For each variable of interest, give sources of data and details of methods of assessment (measurement). Describe comparability of assessment methods if there is more than one group	*4-5*	
Bias	9	Describe any efforts to address potential sources of bias	4-5	
Study size	10	Explain how the study size was arrived at	4	

Continued on next page

For peer review only - http://bmjopen.bmj.com/site/about/guidelines.xhtml

Quantitative variables	11	Explain how quantitative variables were handled in the analyses. If applicable, describe which groupings were chosen and why	4-5	
Statistical methods	12	(*a*) Describe all statistical methods, including those used to control for confounding	5	
		(*b*) Describe any methods used to examine subgroups and interactions	5	
		(*c*) Explain how missing data were addressed	5	
		(*d*) *Cohort study*—If applicable, explain how loss to follow-up was addressed<br>*Case-control study*—If applicable, explain how matching of cases and controls was addressed<br>*Cross-sectional study*—If applicable, describe analytical methods taking account of sampling strategy	4	
		(*e*) Describe any sensitivity analyses	-	
**Results**				
Participants	13*	(a) Report numbers of individuals at each stage of study—eg numbers potentially eligible, examined for eligibility, confirmed eligible, included in the study, completing follow-up, and analysed	6-8	
		(b) Give reasons for non-participation at each stage	4-5	
		(c) Consider use of a flow diagram	-	Not applicable
Descriptive data	14*	(a) Give characteristics of study participants (eg demographic, clinical, social) and information on exposures and potential confounders	6-8	
		(b) Indicate number of participants with missing data for each variable of interest	6-8	
		(c) *Cohort study*—Summarise follow-up time (eg, average and total amount)	4-5; 6-8	
Outcome data	15*	*Cohort study*—Report numbers of outcome events or summary measures over time		
		Case-control study—Report numbers in each exposure category, or summary measures of exposure	*6-8*	
		Cross-sectional study—Report numbers of outcome events or summary measures		
Main results	16	(*a*) Give unadjusted estimates and, if applicable, confounder-adjusted estimates and their precision (eg, 95% confidence interval). Make clear which confounders were adjusted for and why they were included	4-5; 6-8	
		(*b*) Report category boundaries when continuous variables were categorized	4-5; 6-8	
		(*c*) If relevant, consider translating estimates of relative risk into absolute risk for a meaningful time period	-	

Continued on next page

For peer review only

Other analyses	17	Report other analyses done—eg analyses of subgroups and interactions, and sensitivity analyses	-
**Discussion**			
Key results	18	Summarise key results with reference to study objectives	8
Limitations	19	Discuss limitations of the study, taking into account sources of potential bias or imprecision. Discuss both direction and magnitude of any potential bias	11
Interpretation	20	Give a cautious overall interpretation of results considering objectives, limitations, multiplicity of analyses, results from similar studies, and other relevant evidence	8-11
Generalisability	21	Discuss the generalisability (external validity) of the study results	8-11
**Other information**			
Funding	22	Give the source of funding and the role of the funders for the present study and, if applicable, for the original study on which the present article is based	12

*Give information separately for cases and controls in case-control studies and, if applicable, for exposed and unexposed groups in cohort and cross-sectional studies.

Note: An Explanation and Elaboration article discusses each checklist item and gives methodological background and published examples of transparent reporting. The STROBE checklist is best used in conjunction with this article (freely available on the Web sites of PLoS Medicine at http://www.plosmedicine.org/, Annals of Internal Medicine at http://www.annals.org/, and Epidemiology at http://www.epidem.com/). Information on the STROBE Initiative is available at www.strobe-statement.org.

For peer review only

BMJ Open

Exposure to vibrating hand-held tools and biomarkers of nerve injury in plasma - a population-based, observational study

Journal:	*BMJ Open*
Manuscript ID	bmjopen-2022-070450.R1
Article Type:	Original research
Date Submitted by the Author:	16-Feb-2023
Complete List of Authors:	Zimmerman, Malin; Lund University, Department of Translational Medicine; Helsingborg's Hospital, Department of Orthopedics<br>Nilsson, Peter; Lund University, Department of Clinical Sciences Malmö<br>Dahlin, Lars; Lund University, Department of Translational Medicine; Linköping University, Department of Biomedical and Clinical Sciences
**Primary Subject Heading**:	Occupational and environmental medicine
Secondary Subject Heading:	Neurology
Keywords:	Neurophysiology < NEUROLOGY, OCCUPATIONAL & INDUSTRIAL MEDICINE, Neurological injury < NEUROLOGY, Neurological pain < NEUROLOGY

SCHOLARONE™
Manuscripts

I, the Submitting Author has the right to grant and does grant on behalf of all authors of the Work (as defined in the below author licence), an exclusive licence and/or a non-exclusive licence for contributions from authors who are: i) UK Crown employees; ii) where BMJ has agreed a CC-BY licence shall apply, and/or iii) in accordance with the terms applicable for US Federal Government officers or employees acting as part of their official duties; on a worldwide, perpetual, irrevocable, royalty-free basis to BMJ Publishing Group Ltd ("BMJ") its licensees and where the relevant Journal is co-owned by BMJ to the co-owners of the Journal, to publish the Work in this journal and any other BMJ products and to exploit all rights, as set out in our licence.

The Submitting Author accepts and understands that any supply made under these terms is made by BMJ to the Submitting Author unless you are acting as an employee on behalf of your employer or a postgraduate student of an affiliated institution which is paying any applicable article publishing charge ("APC") for Open Access articles. Where the Submitting Author wishes to make the Work available on an Open Access basis (and intends to pay the relevant APC), the terms of reuse of such Open Access shall be governed by a Creative Commons licence – details of these licences and which Creative Commons licence will apply to this Work are set out in our licence referred to above.

Other than as permitted in any relevant BMJ Author's Self Archiving Policies, I confirm this Work has not been accepted for publication elsewhere, is not being considered for publication elsewhere and does not duplicate material already published. I confirm all authors consent to publication of this Work and authorise the granting of this licence.

For peer review only

Exposure to vibrating hand-held tools and biomarkers of nerve injury in plasma

# - a population-based, observational study

Running head: Vibration exposure and nerve biomarkers

Malin Zimmerman MD, PhD[1,2,3], Peter M. Nilsson, MD, PhD[4], Lars B. Dahlin MD, PhD[1,3,5]*

[1] Department of Translational Medicine – Hand Surgery, Lund University, Malmö, Sweden

[2] Department of Orthopedics, Helsingborg Hospital, Helsingborg, Sweden

[3] Department of Hand Surgery, Skåne University Hospital, Malmö, Sweden

[4] Department of Clinical Sciences Malmö, Lund University, Malmö, Sweden

[5] Department of Biomedical and Clinical Sciences, Linköping University, Linköping, Sweden

* Corresponding author:

Lars B. Dahlin, Professor, Department of Translational Medicine – Hand Surgery, Lund University, Jan Waldenströms gata 5, 205 02 Malmö, Sweden; Fax +46 40 928855; lars.dahlin@med.lu.se

Word count: 2196

Keyword: Hand-Arm Vibration Syndrome – peripheral nerve - galanin – nerve degeneration –- neuropathy – biomarkers - vibration – HSP27 – caspase 3 – galectin-3 – Nerve Growth Factor

For peer review only

Abstract

Objectives: To analyze potential biomarkers for vibration-induced nerve damage in a population-based, observational study.

Design: Prospective cohort study.

Setting: Malmö Diet Cancer Study, Malmö, Sweden.

Participants: In a sub-cohort of 3,898 individuals (recruited 1991-1996) from Malmö Diet Cancer Study (MDCS; baseline examination in 28,449 individuals; collection of fasting blood samples in a cardiovascular sub-cohort of MDCS of 5,540 subjects) neuropathy-relevant plasma biomarkers were analyzed during follow up after filling out questionnaires, including a question whether work involved work with vibrating hand-held tools; graded as "not at all", "some" or "much".

Primary outcome measures: The neuropathy-relevant plasma biomarkers VEGF-a, VEGF-d, VEGFR2, galanin, galectin-3 (Gal3), HSP27, ß-NGF, caspase-3, caspase-8, TGF-alpha and TNF were analyzed. Data were analyzed by conventional statistics (Kruskal-Wallis test; posthoc test Mann-Whitney U-test; Bonferroni correction for multiple testing) and in a sub-analysis for galanin using two linear regression models (unadjusted and adjusted).

Results: Among participants, 3,361/3,898 (86%) reported no work with vibrating hand-held tools, 351/3,898 (9%) reported some and 186/3,898 (5%) much work. There were more men and smokers in vibration-exposed groups. Galanin levels were higher after much vibration exposure (arbitrary units 5.16±0.71) compared to no without vibration exposure (5.01±0.76; p=0.015) with no other observed differences.

Conclusions: Higher plasma levels of galanin, possibly related to magnitude, frequency, acceleration, and duration as well as to severity of symptoms of vibration exposure, may be found in individuals working with hand-held vibrating tools.

For peer review only

Strengths and limitations of this study:

- Based on data from the Malmö Diet Cancer Study, neuropathy-relevant plasma biomarkers could be analyzed in a sub-cohort of 3,898 individuals.
- The neuropathy-relevant biomarkers were related to replies from a questionnaire if the participant's work involved exposure to vibrating hand-held tools and graded as "not at all", "some" or "much").
- A wide range of biomarkers were analyzed, consisting of VEGF-a, VEGF-d, VEGFR2, galanin, galectin-3 (Gal3), HSP27, ß-NGF, caspase-3, caspase-8, TGF-alpha and TNF, with focus on galanin; a relevant protein involved in several biological processes, including nociception.
- The generalization of the findings of galanin has in the future to be related to extent of exposure, i.e., various magnitudes, frequencies, accelerations, and durations, in individuals working with vibration-held tools.

For peer review only - http://bmjopen.bmj.com/site/about/guidelines.xhtml

INTRODUCTION

Hand-arm vibration syndrome (HAVS) is a common condition that may induce injuries to a variety of the tissues in the upper extremity, particularly affecting the peripheral nervous and musculoskeletal systems. Approximately 14% of all employed men and 3% of women in Sweden are exposed to vibrations from hand-held tools for at least 25% of their working time.[1] The amount of induced injury to the individual´s extremity depends on the magnitude, frequency and acceleration of the vibration.[2] Vibration exposure may induce the well-known white finger symptoms, but paresthesia, numbness and impaired sensation in the affected extremity, based on an underlaying nerve injury, are probably more common.[3] To confirm a proper HAVS diagnosis, the treating physician must determine a history of vibration exposure, rule out other explaining conditions, and perform the different clinical assessment methods to detect an impaired function.[4] However, there are no specific simple reported biomarkers that can be measured in the blood to support, for example, signs of a neurological injury. The prevalence of vibration-induced neuropathy in HAVS may extend from 7-79% (average 39%).[3] Such symptoms may be caused by structural changes, e.g., extensive axonal degeneration, as observed in biopsies from the posterior interosseous nerve just proximal to the wrist in men exposed to vibrations.[5] Even data from skin biopsies of fingers in vibration-exposed workers indicate that there is a reduction in content of calcitonin gene-related peptide (CGRP) as well as intraepidermal nerve fiber density observed as staining for PGP-9.5 in the biopsies.[6] The structural changes in the peripheral nerve may also induce an increased susceptibility for peripheral nerve compression syndromes, like carpal tunnel syndrome, CTS,[7] in accordance with the relation between diabetic neuropathy and CTS.[8, 9] The aim of the present observational study was to examine several nerve-related biomarkers, as a sign of neuropathy that can be measured in plasma in a cohort of subjects that were exposed to vibrating hand-held tools.

For peer review only

METHODS

Study population

The cohort Malmö Diet Cancer Study (MDCS)[10] consists of 30,446 individuals from the southern part of Sweden. The individuals, living in the city of Malmö and born 1926-1945, were invited to participate in the study during 1991 to 1996. The individuals underwent a health examination, assessment of laboratory tests, and filled in a large number of questionnaires regarding cardiovascular risk factors as previously described in detail [10]. The rate of attendance was approximately 41%, of which 60% of the individuals were women. The characteristics of the cohort, and the inclusion criteria for MDCS have previously been described.[10-14] From the large cohort of 28,449 individuals (11,246 men and 17,203 women) that underwent adequate baseline examination, 6,103 individuals were invited to the specific cardiovascular sub-cohort of the MDCS, where 5,540 subjects accepted inclusion for collection of fasting blood samples. The details of the laboratory analysis were recently presented.[15, 16] In short, blood samples were analyzed using Olink Proseek Multiplex proximity extension assay, which uses two antibodies that bind to the target protein.[16] In the final sub-cohort, 4,865 samples were sent for analysis and after internal quality control 4,741 individuals remained for evaluation of biomarker panels. In 843 individuals, data on vibration exposure was missing, resulting in 3,898 included individuals for the present study.

To analyze vibration exposure, the following question was included in participants´ questionnaires: *"Does your work involve working with vibrating hand-held tools?"* Participants graded their exposure to hand-held vibrating tools as "Not at all", "Some" and "Much", but the duration of exposure or the type of equipment were not defined or reported by the participants. During follow-up, the specific laboratory tests as described were

For peer review only - http://bmjopen.bmj.com/site/about/guidelines.xhtml

performed and the following neuropathy-relevant biomarkers were selected: VEGF-a, VEGF-d, VEGFR2, galanin, galectin-3 (Gal3), HSP27, ß-NGF, caspase-3, caspase-8, TGF-alpha and TNF. Three of these biomarkers have been used in a recent study related to development of the two nerve compression disorders CTS and ulnar nerve entrapment.[15]

Statistical analysis

Nominal data are presented as number (%) and were compared using the Chi square test. The biomarkers are presented as mean ± standard deviation (SD) and are expressed as arbitrary units (AU). The Kruskal-Wallis test was used to analyze potential differences between groups with a Bonferroni correction due to the number of biomarkers (eleven biomarkers; i.e., requiring $p<0.004$). In subsequent Mann-Whitney pairwise comparisons, p-values were also adjusted by the Bonferroni correction for multiple testing. Linear regression models were used to study the effect on vibration exposure on levels of galanin (see results section on reason why galanin was selected). Two models were used, one unadjusted (Model 1) and one adjusted for age at baseline, sex, diabetes and smoking. A p-value of <0.05 was considered significant. IBM SPSS Statistics for Mac version 28.0 (SPSS Inc., Chicago, IL) was used for the statistical analysis.

Patient and Public Involvement statement

No patient, politicians or the public were involved in the development of the research question and the design of the present study. The results of the research, after being published in the scientific journal, will be disseminated in a variety of ways. One way will be information to physicians and surgeons working with patients with HAVS in everyday practice and through the health care organizations connected to small and large companies. Furthermore, the results

For peer review only

will be spread through lectures to other colleagues and students in the health care sector and to any patient organizations or insurance companies with interest in HAVS.

RESULTS

In the total number of individuals, 1,603/3,898 (41%) were men and 2,295/3,898 (59%) were women with a mean age of 57±6 years. At baseline, 3,361/3,898 (86%) reported that they did not at all work with vibrating hand-held tools, while 351/3,898 (9%) and 186/3,898 (5%) reported that their work involved some or much work with vibrating hand-held tools, respectively. There were more men and more smokers in the groups that were exposed to vibrations. The baseline characteristics of the individuals in this study are presented in Table 1.

Table 1. Biomarkers in plasma in workers reporting no, some or much vibration exposure

	**"Does your work involve working with vibrating hand-held tools?"**			
	**Not at all (n=3361)**	**Some (n=351)**	**Much (n=186)**	**P-value**
Men n (%)	1215 (36)	236 (67)	152 (82)	**<0.001**
Age (years)	57±6	58±6	57±6	0.23
Smoking n (%)	860 (26)	107 (30)	63 (34)	**0.009**
Diabetes n (%)	661 (20)	74 (21)	32 (17)	0.56
VEGF-a (n=3898)	10.0±0.49	10.0±0.44	10.1±0.45	0.25
VEGF-d	6.74±0.48	6.70±0.48	6.74±0.57	0.27

For peer review only - http://bmjopen.bmj.com/site/about/guidelines.xhtml

(n=3896)				
VEGFR2	6.86±0.39	6.84±0.39	6.81±0.37	0.27
(n=3788)				
Galanin	5.01±0.76	5.08±0.77	5.16±0.71	**0.003***
(n=3898)				
Galectin-3 (Gal3)	5.15±0.42	5.10±.44	5.16±0.37	0.12
(n=3898)				
HSP27	4.76±0.84	4.79±0.80	4.84±0.78	0.46
(n=3897)				
ß-NGF	1.18±0.39	1.36±0.67	1.12±0.25	0.38
(n=403)				
Caspase-3	10.74±0.95	10.80±0.90	10.79±0.93	0.52
(n=3718)				
Caspase-8	1.58±0.69	1.64±0.72	1.70±0.74	0.10
(n=3865)				
TGF-alpha	1.39±0.34	1.38±0.33	1.36±0.32	0.31
(n=3786)				
TNF	1.33±1.68	1.20±1.95	2.18±3.1	0.79
(n=128)				

Biomarkers presented as mean level of AU: arbitrary units ± standard deviation (SD). Statistical analysis based on Chi-Square (sex, smoking and diabetes) or Kruskal Wallis test; requiring $p<0.004$ (eleven biomarkers). * In subsequent Mann-Whitney pairwise comparisons the statistically significant difference was found between "not at all" and "much" with a p-value of 0.015 adjusted by the Bonferroni correction for multiple tests.

Biomarkers

Observed differences in biomarker plasma levels were small (Table 1). Galanin levels were higher in the group reporting much vibration exposure compared to the group without

For peer review only

vibration exposure (Table 1). No other differences among the other analyzed biomarkers were observed. When further analyzing galanin levels, being in the group that reported high vibration exposure predicted higher levels of galanin in the unadjusted model (Table 2). When adjusting for age, sex, smoking and diabetes, no significant predictions could be made in the regression model (Table 2).

Table 2. Linear regression models of galanin levels in plasma **in workers reporting no, some or much vibration exposure**

	Model 1	Model 2
No vibration exposure	Reference	Reference
Some vibration exposure	0.06 (-0.02-0.15)	-0.04 (-0.12-0.04)
Much vibration exposure	0.14 (0.03-0.26)	-0.02 (-0.13-0.09)
Age		-0.01 (-0.01- -0.01)
Sex (female reference)		0.41 (0.36-0.46)
Smoking		-0.32 (-0.37- -0.27)
Diabetes		-0.13 (-0.19- -0.08)

Unadjusted B-coefficients with 95% confidence interval. Model 1 unadjusted and model 2 adjusted for age, sex, smoking and diabetes.

DISCUSSION

The present population-based, observational study indicates that individuals with exposure to vibrating hand-held tools in a large cohort of around 30,000 patients, where levels of several biomarkers related to nerves, and possible vibration-induced neuropathy, were available in around 3898 individuals with also information about exposure to vibrating hand-held tools, showed increased levels of plasma galanin. In contrast, other relevant nerve-related biomarkers, including the neuroprotective substance HSP27, which is relevant in diabetic

For peer review only - http://bmjopen.bmj.com/site/about/guidelines.xhtml

For peer review only

neuropathy[17, 18] and nephropathy,[19] did not show any statistically significant alterations among the vibration-exposed individuals.

Galanin is involved in several biological processes,[16, 20] including nociceptive sensory processing in the spinal cord. Galanin and Neuropeptide Y are consistently upregulated on both gene and protein levels in dorsal root ganglia in rodents with a potential relation to pain after a severe nerve injury;[21-23] a symptom which may also be present among individuals with HAVS.[24, 25] Experimental studies in rats also indicate that a sciatic nerve compression, which is a less pronounced injury, increases the number of galanin-stained sensory neurons in dorsal root ganglia (DRG) compared to uninjured and contralateral sensory neurons.[26] Furthermore, a complete nerve transection is more efficient than a partial nerve transection or a slight compression injury as an inducer of galanin expression in DRG sensory neurons.[26] Such injuries seem to induce an up-regulation of galanin in mainly small diameter sensory neurons and to a lesser degree in larger neurons.[26] Thus, the extent of induction of galanin expression is related to the severity of nerve injury.[27] Interestingly, the induction of galanin in small diameter neurons in the dorsal root ganglia, which are related to smaller diameter nerve fibers in the sciatic nerve, is notable, since small diameter nerve fibers are reported to be affected by experimental vibration exposure in rat paws.[28] The structural changes seem to occur close to the source of vibration exposure and not more proximally.[28] Whether the present increase in galanin is due to up-regulation of galanin in small or large diameter nerve fibers is not possible to analyze in our cohort. However, our results indicate that a self-reported history of slight or moderate exposure to vibrating hand-held tools is sufficient to provoke a nerve injury that results in increased, and persistent, plasma levels of galanin. On the other hand, it is not surprising that for example beta-NGF did not show any increased plasma levels, since such a substance is only transiently up-regulated

For peer review only - http://bmjopen.bmj.com/site/about/guidelines.xhtml
For peer review only

after a severe nerve injury (i.e., nerve transection)[29] and vibration-exposure may hence not be reflected by any change in beta-NGF in plasma; which is probably also applicable for some of the other biomarkers. Pro- (e.g., TNF) and anti-inflammatory (TGF-ß) cytokines are expressed after Wallerian degeneration, where the former promotes recruitment of macrophages, and the latter are expressed after macrophage recruitment with a task to attenuate inflammation.[30] The present vibration exposure was not severe enough to induce a nerve injury resulting in higher levels of such cytokines among the participants with much vibration exposure. Another relevant protein is Galectin-3, which is considered to modulate cell to extracellular matrix interactions, but also as a mediator of signal transduction event on the cell surface as well as a variety of extracellular processes.[31] This multicellular protein was selected to be analyzed in relation to vibration exposure since one suspect it may be increased in view of described structural changes in affected nerves. However, the plasma levels were not different between the participants with and without vibration exposure.

Thus, we did not find any increased plasma levels of the other measured biomarkers. The caspases indicate apoptosis of, for example, Schwann cells that occur in a peripheral nerve after injury in experimental systems.[32] A more severe experimental nerve injury may also induce increased up-regulation of HSP27 locally in the peripheral nerve as well as in sensory neurons.[33] Furthermore, a generalized neuropathy, such as diabetic neuropathy, increases plasma levels of HSP27, with an association between plasma levels and protection of nerve function.[17, 18] Other neuropathies, including diabetic neuropathy, also increase a variety of plasma proteins that indicate affection and injury to the nerve fibers, i.e., axons and Schwann cells, as well as to vascular components.[34-36] However, we did not find any increased plasma levels of these biomarkers in the present cohort. There are also reports that there is a significantly altered expression of insulin growth factor-1 (IGF-1) immunoreactivity in

For peer review only - http://bmjopen.bmj.com/site/about/guidelines.xhtml

tendons and in nerves after experimental vibration exposure.[37, 38] VEGF and its receptors may also be of relevance since they have been implicated in treatment of nerve regeneration[39] and in diabetic neuropathy.[40] This factor, initially defined as being angiogenetic, is described as relevant in the cross-talk between peripheral nerve components and blood vessels;[41] again being of potential interest in HAVS due to the vascular and neural component in the syndrome. However, even the participants opinion of much vibration exposure was not sufficient to increase the plasma levels. Thus, a number of potentially relevant biomarkers were selected and analyzed, but were not found to be high in the plasma. Recently, low molecular organic biomarkers were analyzed in blood samples in vibration exposed workers using metabolomics, showing differences in those with vibration-induced white fingers.[42] However, these metabolites were not possible to analyze in the present cohort. The interpretation of the present results is that vibration exposure is not sufficient to constantly increase any up-regulation of a variety of biomarkers, except galanin, in the peripheral nerve that is mirrored in the plasma levels.

Strengths and limitations

The percentage of participants that reported exposure to hand-held vibrating tools was 14%, which is slightly lower than official data in Sweden.[43] Thus, it is reported that 19% of the population (all type of professions) work with hand-held machines at least 25% of the working time 2017-2019 (interpreted to be relatively unchanged during the last decades) with a difference between men (25%) and women (12%) .[44] Part of the difference may be due to the specific question asked in the present study (*Does your work involve working with vibrating hand-held tools?"*), which could by the participants be interpreted as both a limited as well as an extensive exposure. In addition, since the question was referring to "vibrating hand-held tools", we anticipate that the vibrations were of certain frequencies and most

For peer review only

probably not very low frequencies. We had no information about disease or severity stages, e.g., the Stockholm scales, which is a limitation. A further limitation of the study is that we had no detailed data on the exact level of vibration exposure for each participant, such as cumulative exposure (i.e., magnitude, frequency, and acceleration), which would have enabled more detailed analysis with prediction of risk for a higher level of galanin in relation to cumulative exposure. A specified analysis of cumulative exposure requires detailed analysis of each participant with preferably measurements at the working place and related to their profession. However, the strengths include the well-defined cohort and the large study sample.

CONCLUSIONS

We conclude that higher levels of plasma galanin may be found in individuals reporting work with vibrating hand-held tools. Future research should investigate whether galanin might be useful as a biomarker in HAVS in relation to vibration exposure at various magnitudes, frequencies, accelerations, and durations as well as to severity of symptoms.

For peer review only

STATEMENTS

Contributorship

A significant contribution was made by all authors stated in this article. MZ , PN and LD designed the study, the first draft was made by MZ and LD and all authors contributed to the interpretation of the data. Finally, all authors reviewed and accepted the final version before publishing.

Funding statement

This work was supported by grants from Region Skåne, Lund University, Kockska stiftelsen, Stig and Ragna Gorthon Foundation, The Swedish Diabetes Foundation [DIA2020-492], the Swedish Research Council [2022-01942], the Regional Agreement on Medical Training and Clinical Research (ALF) between Region Skåne and Lund University and Magnus Bergvall foundation [2020-03612]. The sponsors had no role in the design of the study, collection, analysis, and interpretation of data, writing of the report or submission of the paper.

Competing of Interests

Authors declare no conflict of interest.

Ethics approval

This project has been approved by the Regional Ethical Review Board in Lund, Sweden (51-90, 2009-633, 2011-356, 2014-742, 2015-686, 2015-754, 2016-479) and by the Swedish Ethical Review Authority (2019-01439). All participants gave a signed informed consent to participate in the cohort.

Data sharing

For peer review only

Public access to data is restricted by the Swedish Authorities (Public Access to Information and Secrecy Act; https://government.se/information-material/2009/09/public-access-toinformation-andsecrecy-act/), but data can be made available for researchers after a special review that includes approval of the research project by both the national Swedish Ethical Review Authority and the authorities' data safety committees.

Acknowledgements

The authors are very grateful to all the participants in MDCS whose participation enabled this work. We are also grateful to Anders Dahlin for his help with data extraction and Tina Folker for her administrative assistance.

For peer review only

References

1. Swedish Work Environment Authority. The Work Environment Act. 2014.
2. Griffin MJ. Measurement, evaluation, and assessment of occupational exposures to hand-transmitted vibration. *Occup Environ Med*. 1997;54:73-89.
3. Nilsson T, Wahlstrom J, Burstrom L. Hand-arm vibration and the risk of vascular and neurological diseases-A systematic review and meta-analysis. *PLoS One*. 2017;12:e0180795.
4. Shen SC, House RA. Hand-arm vibration syndrome: What family physicians should know. *Can Fam Physician*. 2017;63:206-210.
5. Stromberg T, Dahlin LB, Brun A, Lundborg G. Structural nerve changes at wrist level in workers exposed to vibration. *Occup Environ Med*. 1997;54:307-311.
6. Goldsmith PC, Molina FA, Bunker CB, et al. Cutaneous nerve fibre depletion in vibration white finger. *J R Soc Med*. 1994;87:377-381.
7. Dahlin LB, Sanden H, Dahlin E, Zimmerman M, Thomsen N, Bjorkman A. Low myelinated nerve-fibre density may lead to symptoms associated with nerve entrapment in vibration-induced neuropathy. *J Occup Med Toxicol*. 2014;9:7.
8. Thomsen NO, Mojaddidi M, Malik RA, Dahlin LB. Reduced myelinated nerve fibre and endoneurial capillary densities in the forearm of diabetic and non-diabetic patients with carpal tunnel syndrome. *Acta Neuropathol*. 2009;118:785-791.
9. Zimmerman M, Gottsater A, Dahlin LB. Carpal Tunnel Syndrome and Diabetes-A Comprehensive Review. *J Clin Med*. 2022;11:1674.
10. Berglund G, Elmstahl S, Janzon L, Larsson SA. The Malmo Diet and Cancer Study. Design and feasibility. *J Intern Med*. 1993;233:45-51.

For peer review only

11. Rydberg M, Zimmerman M, Lofgren JP, et al. Metabolic factors and the risk of Dupuytren's disease: data from 30,000 individuals followed for over 20 years. *Sci Rep*. 2021;11:14669.

12. Hedblad B, Nilsson P, Janzon L, Berglund G. Relation between insulin resistance and carotid intima-media thickness and stenosis in non-diabetic subjects. Results from a cross-sectional study in Malmo, Sweden. *Diabet Med*. 2000;17:299-307.

13. Manjer J, Carlsson S, Elmstahl S, et al. The Malmo Diet and Cancer Study: representativity, cancer incidence and mortality in participants and non-participants. *Eur J Cancer Prev*. 2001;10:489-499.

14. Xue L, Borne Y, Mattisson IY, et al. FADD, Caspase-3, and Caspase-8 and Incidence of Coronary Events. *Arterioscler Thromb Vasc Biol*. 2017;37:983-989.

15. Bergsten E, Rydberg M, Dahlin LB, Zimmerman M. Carpal Tunnel Syndrome and Ulnar Nerve Entrapment at the Elbow Are Not Associated With Plasma Levels of Caspase-3, Caspase-8 or HSP27. *Front Neurosci*. 2022;16:809537.

16. Korduner J, Nilsson PM, Melander O, et al. Proteomic and Metabolomic Characterization of Metabolically Healthy Obesity: A Descriptive Study from a Swedish Cohort. *J Obes*. 2021;2021:6616983.

17. Pourhamidi K, Dahlin LB, Boman K, Rolandsson O. Heat shock protein 27 is associated with better nerve function and fewer signs of neuropathy. *Diabetologia*. 2011;54:3143-3149.

18. Pourhamidi K, Skarstrand H, Dahlin LB, Rolandsson O. HSP27 concentrations are lower in patients with type 1 diabetes and correlate with large nerve fiber dysfunction. *Diabetes Care*. 2014;37:e49-50.

For peer review only

19. Jakhotia S, Sivaprasad M, Shalini T, et al. Circulating levels of Hsp27 in microvascular complications of diabetes: Prospects as a biomarker of diabetic nephropathy. *J Diabetes Complications*. 2018;32:221-225.

20. Ullrich D, Mac Gillavry DW. Mini-review: A possible role for galanin in post-traumatic stress disorder. *Neurosci Lett*. 2021;756:135980.

21. Chalaki M, Cruz LJ, van Neerven SGA, Verhaagen J, Dahan A, Malessy MJA. Molecular Changes in the Dorsal Root Ganglion during the Late Phase of Peripheral Nerve Injury-induced Pain in Rodents: A Systematic Review. *Anesthesiology*. 2022;136:362-388.

22. Shi TJ, Tandrup T, Bergman E, Xu ZQ, Ulfhake B, Hokfelt T. Effect of peripheral nerve injury on dorsal root ganglion neurons in the C57 BL/6J mouse: marked changes both in cell numbers and neuropeptide expression. *Neuroscience*. 2001;105:249-263.

23. Zhang X, Xu ZO, Shi TJ, et al. Regulation of expression of galanin and galanin receptors in dorsal root ganglia and spinal cord after axotomy and inflammation. *Ann N Y Acad Sci*. 1998;863:402-413.

24. Alvarez P, Bogen O, Levine JD. Nociceptor Interleukin 33 Receptor/ST2 Signaling in Vibration-Induced Muscle Pain in the Rat. *J Pain*. 2020;21:506-512.

25. House R, Wills M, Liss G, Switzer-McIntyre S, Manno M, Lander L. Upper extremity disability in workers with hand-arm vibration syndrome. *Occup Med (Lond)*. 2009;59:167-173.

26. Dahlin LB, Stenberg L, Kanje M. Galanin expression in sensory neurons after nerve compression or transection. *Neuroreport*. 2003;14:359-362.

27. Ma W, Bisby MA. Differential expression of galanin immunoreactivities in the primary sensory neurons following partial and complete sciatic nerve injuries. *Neuroscience*. 1997;79:1183-1195.

For peer review only - http://bmjopen.bmj.com/site/about/guidelines.xhtml

28. Lundborg G, Dahlin LB, Hansson H-A, Kanje M, Necking LE. Vibration exposure and peripheral nerve fiber damage. *J Hand Surg*. 1990;15A:346-351.

29. Shakhbazau A, Martinez JA, Xu QG, Kawasoe J, van Minnen J, Midha R. Evidence for a systemic regulation of neurotrophin synthesis in response to peripheral nerve injury. *J Neurochem*. 2012;122:501-511.

30. Fregnan F, Muratori L, Simoes AR, Giacobini-Robecchi MG, Raimondo S. Role of inflammatory cytokines in peripheral nerve injury. *Neural Regen Res*. 2012;7:2259-2266.

31. Ochieng J, Furtak V, Lukyanov P. Extracellular functions of galectin-3. *Glycoconj J*. 2002;19:527-535.

32. Stenberg L, Dahlin LB. Gender differences in nerve regeneration after sciatic nerve injury and repair in healthy and in type 2 diabetic Goto-Kakizaki rats. *BMC Neurosci*. 2014;15:107.

33. Stenberg L, Hazer Rosberg DB, Kohyama S, Suganuma S, Dahlin LB. Injury-Induced HSP27 Expression in Peripheral Nervous Tissue Is Not Associated with Any Alteration in Axonal Outgrowth after Immediate or Delayed Nerve Repair. *Int J Mol Sci*. 2021;22.

34. Rossor AM, Reilly MM. Blood biomarkers of peripheral neuropathy. *Acta Neurol Scand*. 2022;146:325-331.

35. Notturno F, Capasso M, DeLauretis A, Carpo M, Uncini A. Glial fibrillary acidic protein as a marker of axonal damage in chronic neuropathies. *Muscle Nerve*. 2009;40:50-54.

36. Maalmi H, Strom A, Petrera A, et al. Serum neurofilament light chain: a novel biomarker for early diabetic sensorimotor polyneuropathy. *Diabetologia*. 2023;66:579-589.

For peer review only

37. Hansson HA, Dahlin LB, Löwenadler B, Lundborg G, Paleus S, Skottner A. Transient increase in insulin-like growth factor 1 immunoreactivity in rat peripheral nerves exposed to vibrations. *Acta Phys Scand*. 1988;132:35-41.

38. Hansson HA, Dahlin LB, Lundborg G, Löwenadler B, Paleus S, Skottner A. Transiently increased insulin-like growth factor 1 immunoreactivity in tendons after vibration trauma. *Scand J Plast Reconstr Surg Hand Surg*. 1988;22:1-6.

39. Fu C, Hong G, Wang F. Favorable effect of local VEGF gene injection on axonal regeneration in the rat sciatic nerve. *J Huazhong Univ Sci Technolog Med Sci*. 2007;27:186-189.

40. Mojaddidi MA, Ahmed MS, Ali R, et al. Molecular and pathological studies in the posterior interosseous nerve of diabetic and non-diabetic patients with carpal tunnel syndrome. *Diabetologia*. 2014;57:1711-1719.

41. Ruiz de Almodovar C, Lambrechts D, Mazzone M, Carmeliet P. Role and therapeutic potential of VEGF in the nervous system. *Physiol Rev*. 2009;89:607-648.

42. Vihlborg P, Graff P, Hagenbjork A, Hadrevi J, Bryngelsson IL, Eriksson K. Serum Metabolites in Hand-Arm Vibration Exposed Workers. *J Occup Environ Med*. 2020;62:460-465.

43. Arbetsmiljöverket. Arbetsmiljöstatistik Rapport 2020:2. 2020.

44. Arbetsmiljöverket. Arbetsmiljöstatistik Rapport 2020:2; tabellbilaga. 2020.

For peer review only - http://bmjopen.bmj.com/site/about/guidelines.xhtml

STROBE Statement—checklist of items that should be included in reports of observational studies

	Item No.	Recommendation	Page No.	Relevant text from manuscript
**Title and abstract**	1	(*a*) Indicate the study's design with a commonly used term in the title or the abstract	1	
		(*b*) Provide in the abstract an informative and balanced summary of what was done and what was found	2	
**Introduction**				
Background/rationale	2	Explain the scientific background and rationale for the investigation being reported	3	
Objectives	3	State specific objectives, including any prespecified hypotheses	3	
**Methods**				
Study design	4	Present key elements of study design early in the paper	4	
Setting	5	Describe the setting, locations, and relevant dates, including periods of recruitment, exposure, follow-up, and data collection	4-5	
Participants	6	(*a*) *Cohort study*—Give the eligibility criteria, and the sources and methods of selection of participants. Describe methods of follow-up<br>*Case-control study*—Give the eligibility criteria, and the sources and methods of case ascertainment and control selection. Give the rationale for the choice of cases and controls<br>*Cross-sectional study*—Give the eligibility criteria, and the sources and methods of selection of participants	4-5	
		(*b*) *Cohort study*—For matched studies, give matching criteria and number of exposed and unexposed<br>*Case-control study*—For matched studies, give matching criteria and the number of controls per case		
Variables	7	Clearly define all outcomes, exposures, predictors, potential confounders, and effect modifiers. Give diagnostic criteria, if applicable	4-5	
Data sources/ measurement	8*	For each variable of interest, give sources of data and details of methods of assessment (measurement). Describe comparability of assessment methods if there is more than one group	*4-5*	
Bias	9	Describe any efforts to address potential sources of bias	4-5	
Study size	10	Explain how the study size was arrived at	4	

Continued on next page

For peer review only

Quantitative variables	11	Explain how quantitative variables were handled in the analyses. If applicable, describe which groupings were chosen and why	4-5	
Statistical methods	12	(*a*) Describe all statistical methods, including those used to control for confounding	5	
		(*b*) Describe any methods used to examine subgroups and interactions	5	
		(*c*) Explain how missing data were addressed	5	
		(*d*) *Cohort study*—If applicable, explain how loss to follow-up was addressed<br>*Case-control study*—If applicable, explain how matching of cases and controls was addressed<br>*Cross-sectional study*—If applicable, describe analytical methods taking account of sampling strategy	4	
		(*e*) Describe any sensitivity analyses	-	
**Results**				
Participants	13*	(a) Report numbers of individuals at each stage of study—eg numbers potentially eligible, examined for eligibility, confirmed eligible, included in the study, completing follow-up, and analysed	6-8	
		(b) Give reasons for non-participation at each stage	4-5	
		(c) Consider use of a flow diagram	-	Not applicable
Descriptive data	14*	(a) Give characteristics of study participants (eg demographic, clinical, social) and information on exposures and potential confounders	6-8	
		(b) Indicate number of participants with missing data for each variable of interest	6-8	
		(c) *Cohort study*—Summarise follow-up time (eg, average and total amount)	4-5; 6-8	
Outcome data	15*	*Cohort study*—Report numbers of outcome events or summary measures over time		
		Case-control study—Report numbers in each exposure category, or summary measures of exposure	*6-8*	
		Cross-sectional study—Report numbers of outcome events or summary measures		
Main results	16	(*a*) Give unadjusted estimates and, if applicable, confounder-adjusted estimates and their precision (eg, 95% confidence interval). Make clear which confounders were adjusted for and why they were included	4-5; 6-8	
		(*b*) Report category boundaries when continuous variables were categorized	4-5; 6-8	
		(*c*) If relevant, consider translating estimates of relative risk into absolute risk for a meaningful time period	-	

Continued on next page

For peer review only

Other analyses	17	Report other analyses done—eg analyses of subgroups and interactions, and sensitivity analyses	-
**Discussion**			
Key results	18	Summarise key results with reference to study objectives	8
Limitations	19	Discuss limitations of the study, taking into account sources of potential bias or imprecision. Discuss both direction and magnitude of any potential bias	11
Interpretation	20	Give a cautious overall interpretation of results considering objectives, limitations, multiplicity of analyses, results from similar studies, and other relevant evidence	8-11
Generalisability	21	Discuss the generalisability (external validity) of the study results	8-11
**Other information**			
Funding	22	Give the source of funding and the role of the funders for the present study and, if applicable, for the original study on which the present article is based	12

*Give information separately for cases and controls in case-control studies and, if applicable, for exposed and unexposed groups in cohort and cross-sectional studies.

Note: An Explanation and Elaboration article discusses each checklist item and gives methodological background and published examples of transparent reporting. The STROBE checklist is best used in conjunction with this article (freely available on the Web sites of PLoS Medicine at http://www.plosmedicine.org/, Annals of Internal Medicine at http://www.annals.org/, and Epidemiology at http://www.epidem.com/). Information on the STROBE Initiative is available at www.strobe-statement.org.

For peer review only
